# How is who: evidence as clues for action in participatory sustainability science and public health research

**DOI:** 10.1007/s40656-023-00603-5

**Published:** 2024-01-09

**Authors:** Guido Caniglia, Federica Russo

**Affiliations:** 1 Konrad Lorentz Institute, Klosterneuburg, Austria; 2 Utrecht University, Utrecht, The Netherlands; 3 University College London, London, UK

**Keywords:** Sustainability science, Public health, Evidence, Susan Haack, Mixed mechanisms, Transdisciplinary research, Partecipatory research

## Abstract

Participatory and collaborative approaches in sustainability science and public health research contribute to co-producing evidence that can support interventions by involving diverse societal actors that range from individual citizens to entire communities. However, existing philosophical accounts of evidence are not adequate to deal with the kind of evidence generated and used in such approaches. In this paper, we present an account of *evidence as clues for action* through participatory and collaborative research inspired by philosopher Susan Haack’s theory of evidence. Differently from most accounts of evidence for use in policies and interventions, our account combines action-oriented (the how) and actors-oriented (the who) considerations. We build on Haack’s theory and on the analysis of examples of participatory and collaborative research in sustainability science and public health research to flesh out six procedural criteria for the generation and mobilization of evidence in and from participatory research. Action-oriented criteria invite to look at evidence from a (a) foundherentist, (b) gradational and (c) quasi-holistic perspective. Actors-oriented criteria point out that evidence generation and utilization are (d) social, (e) personal, and (f) embedded. We suggest that these criteria may reinforce participatory and collaborative approaches to evidence co-production when addressing complex problems in sustainability science and public health allowing for the generation of a kind of practical objectivity.

## Introduction

Sustainability science and public health research deal with interconnected real-world challenges of our time, such as loss of biodiversity and food security or health disparities in responding to epidemics and pandemics (IPCC 2023). These challenges regard wide sectors of the population and disproportionately affect the most vulnerable, for example those disadvantaged due to, among others, race, ethnicity, income, age, ability or gender (Djoudi et al., 2016; Kosanic et al., 2022; Valles, 2018; Venkatapuram, 2011). Sustainability science and public health research attempt to generate or mobilize evidence that can serve, for instance, to design interventions and programs that address these challenges at different scales as well as to contribute to crafting and implementing new and more effective policies (Parkhurst, 2016; Sterner et al., 2019).

In recent years, numerous critiques have been moved to programs that base policies and decisions solely on evidence generated through research inspired by Evidence-Based Medicine (EBM) and Randomized Controlled Trials (RCTs) (Montuschi, 2017; Parkhurst, 2016). Some of these critiques have pointed out that evidence-based approaches adhere to a paradigm of unrealistic naïve rationality or that their reliance on RCTs leaves out important pieces of evidence (Parkkinen et al., 2018). Others have defined appeals to evidence-based policy, solutions, and interventions as a myth of modernist societies (Hammersley, 2005; Stirling, 2010). In sustainability science, for example, appeals to evidence, such as when talking about evidence-based solutions to climate change, have often been criticized for not capturing the ambiguities, contradictions, tensions and conflicts of the social, cultural, and political dynamics involved (Reed & Meagher, 2019; Sanderson, 2006). In public health research, the use of methodological hierarchies for the generation and evaluation of evidence has been criticized for not taking into consideration the importance of context or the specific purposes of interventions and their appropriateness (Dobrow et al., 2004). Overall, works from multiple disciplines have critically reflected on the way evidence is used in policy-, decision-making, and action-contexts where the issues at hand are rather practical (as different to factual), multiple factors (from local to ideological) need consideration, and many actors with conflicting perspectives and interests are involved (Cairney & Oliver, 2017; Cartwright & Hardie, 2012; Parkhurst, 2016; Sanderson, 2002).

A variety of participatory and collaborative approaches have emerged that can help to overcome these critiques. First, these approaches are participatory, as they include actors outside of academia directly affected by environmental and health problems by foregrounding issues of environmental and health equity and justice. Second, they are also collaborative, because they engage in knowledge co-production processes towards the empowerment of those actors (Freudenberg & Tsui, 2014; Wyborn et al., 2019). Philosophers of science have been increasingly engaging with forms of participatory and collaborative research both in sustainability and health contexts (Eigi-Watkin & Koskinen, 2023; Evans & Potochnik, 2020; Ludwig & El-Hani, 2020; Ludwig et al., 2021). Further, examples such as the Multiple Evidence Base approach in sustainability science (MEB in Box 1) or Community Based Participatory Research in public health (CBPR in Box 2) have attracted increasing attention because of their ability to develop evidence about the complexity of sustainability and health problems (Menatti et al., 2022) as well as about the appropriateness and meaningfulness of interventions (Leask et al., 2019; Reed & Meagher, 2019).

Yet, existing philosophical accounts of evidence for policies, evidence for use or evidence-based practice (Cartwright & Hardie, 2012; Game et al., 2018) do not fit the processes and insights of participatory and collaborative work supporting decision-making and action for two reasons. On the one hand, even if some of these accounts have stressed the evidential role of mechanisms (the how), they tend to overlook the importance of a multiplicity of social, cultural and political factors that need to be taken into considerations and leveraged (Kelly & Russo, 2018, 2021; Kelly et al., 2014). On the other, they do not capture the uncertain and often-contentious processes that allow multiple actors, especially those most affected by sustainability and health problems, to contribute to addressing such problems (the who) (Meyer & Northridge, 2007; Reed & Meagher, 2019).

Differently, we argue that an account of evidence for action needs to take into consideration both the how (from design to implementation and evaluation) and the who (actors). When talking about evidence for action we refer to the clues that emerge from participatory and collaborative processes of co-production and that may inform the shared design, enactment and implementation of interventions and processes, such as new or ongoing interventions, policies and programs. Central to this definition is the notion of ‘clue’ that we borrow from philosopher Susan Haack (Haack, 1993, 2001, 2014). By characterizing *evidence as clues*, we emphasize that evidence (i) does not provide certainty, (ii) is often contextual, (iii) suggests directions for further inquiry, and (iv) can be given meaning within complex arguments and reasoning often involving a variety of kinds of knowledge and ways of knowing (from those of science to those of local communities). Thinking of evidence as clues means to acknowledge that although evidence is not technical, formal, value-free or neutral, it can still provide practical guidance and support for some claim or intervention. This idea of support has gone lost in dominant evidence-based approaches, in which evidence has taken instead connotations of certainty, predictability, and control. Haack’s theory of evidence as clues helps to regain the idea of evidence as support and to identify procedural criteria that can both describe and normatively guide processes of evidence co-production. We present six main criteria and argue that using them may support the generation of practical objectivity supporting decision-making and action processes (Montuschi, 2017).

In the following, first, we introduce participatory and collaborative approaches in sustainability science and public health referring to MEB and CBPR as examples of evidence co-production (also Box 1 and Box 2). Second, we suggest that Susan Haack’s theory of evidence as clues can help to make sense of evidencing processes in such approaches. Then, we present the six procedural criteria that emerge from Haack’s work and allow for rethinking the way evidence is addressed in participatory research. Further, we exemplify and detail how these criteria can help to make sense of how evidence is generated and mobilized in specific studies utilizing MEB or CBPR. We discuss the vantage point of looking at evidence for action as clues through the six procedural criteria and conclude by reiterating the need to develop understandings of evidence that account for the complexities of the problems addressed in and of the processes of participatory and collaborative sustainability and public health research.

## Participatory and collaborative knowledge co-production in sustainability science and public health research

Public participation in science and bioscience, from environmental to health sciences, has become a matter of growing importance (Kelty & Panofsky, 2014; Kelty et al., 2014). Participatory approaches vary greatly across domains and methodologies connecting to different communities and histories of social movements and activism (Buyx et al., 2017; Hess, 2012). There are many different ways of understanding participation in relation to research processes, from patients taking part to study trials to citizens contributing their knowledge to existing scientific endeavor or non-academic actors actively contributing to shaping new research questions and topics. Importantly, there are different degrees of collaborative engagement, from helicopter science (with no contribution to the design of participants) to highly collaborative and interactive research approaches that conceive of researchers as partners on equal footing (Balazs & Morello-Frosch, 2013). In this paper, we deal with approaches that aim to co-produce knowledge and evidence by involving vulnerable and marginalized communities (most affected by health and environmental threats) through collaborative processes that see all partners as contributing on equal footing (Evans & Potochnik, 2020; Ludwig et al., 2021).

Sustainability science and public health research have witnessed an increasing recognition of the role of participatory and collaborative approaches. These research formats include academics, practitioners, decision-makers and those directly affected by the problems that need to be addressed. They have a justice orientation, which relies on the idea that direct involvement of those most affected by environmental and health injustices are the ones entitled to provide knowledge and solutions about how to overcome such injustices (Israel et al., 2013; Minkler et al., 2010; Tengö et al., 2014). They support decisions that are not only based on technical experts’ knowledge (such as medical-epidemiological in public health or engineering and solutions in sustainability science), but consider the broad range of social and political factors, tensions and contingencies that influence health (e.g., social determinants of heath) and sustainability (e.g., socio-political dimensions of sustainability) (Fairchild et al., 2010; Stirling, 2010).

In sustainability science, these approaches often go under the name of transdisciplinary sustainability science (Lang et al., 2012), action-oriented sustainability research (Caniglia et al., 2021; Fazey et al., 2018), and knowledge co-production (Chambers et al., 2021; Wyborn et al., 2019). From the many existing approaches, the Multiple Evidence Base approach (MEB) (Box 1) exemplifies how Indigenous or local communities have been involved in research related to the management of biodiversity and common resources also towards human wellbeing (Malmer et al., 2020; Tengö et al., 2014). In public health, different forms of community-based, participatory, and action-oriented research have also engaged with marginalized groups (e.g., disadvantaged women or people from gender and sexual minorities), for example to prevent chronic diseases and obesity or to address the health impacts of social isolation and the spread of diseases (Cusworth et al., 2015; Echo-Hawk, 2011; Meyer & Northridge, 2007; Walls et al., 2022). An umbrella term often used to refer to these approaches in public health is Community Based Participatory Research (CBPR) (see Box 2) (Israel et al., 2013; Minkler et al., 2010).

**Table:** 

The Multiple Evidence Based Approach (MEB)	
The **Multiple Evidence Based Approach** interweaves indigenous, local and scientific knowledge systems in order to enhance our understanding of governance of biodiversity, ecosystems, and more generally of common resources, for human well-being (Tengö et al., 2014, 2017). The approach has been used in numerous action-oriented, participatory and collaborative research projects and programs (Malmer et al., 2020), from the development of sustainable management of common resources on Indigenous and aboriginal land (Robinson et al., 2015) to the development of conservation strategies for pollinators in the context of pollinators’ decline (Smith et al., 2017) (see Sect. 7). The MEB has been included in international biodiversity assessment programs such as the Intergovernmental Science-Policy Platform on Biodiversity and Ecosystem Services (IPEBS) (Tengö et al., 2014) ***Collaboration and co-creation*:** The MEB understands knowledge systems as composed of actors, practices and institutions that organize the production, transfer and use of knowledge. It looks at indigenous, local and scientific knowledge systems as complementary, which implies the need to maintain their integrity and foster collaboration across knowledge holders and experts (e.g., members from Indigenous populations). The MEB encourages cross-fertilization “grounding collaboration on an equal starting point whereas contributors define the goal of the collaboration and mutually agreed ways to proceed” (Tengö et al., 2014, p. 548). In assessment programs (IPEBS), for example, this means: (1) defining problems and goals in collaborative manner, (2) generating an enriched picture of problems and goals drawing on an agreed upon diversity of knowledge, (3) reflection on social and environmental implications of results. ﻿ ***Evidencing process*:** The processes of knowledge validation and evaluation is central to the MEB. In order to build evidence for ecosystem governance, the MEB recognizes that each of the knowledge systems should “speak for itself”, within its own context and with its own criteria, without assigning one knowledge system (such as the Western science one) the role of external validator: “Processes of validating knowledge need to recognize and respect differences in theoretical and methodological approaches to understanding the biophysical world as well as underlying world-views” (Tengö et al., 2014). It is on these bases that it may be possible to interweave internally-validated knowledge for the governance of environmental commons by: *mobilizing* (to allow for sharing), *translating* (to enable mutual comprehension), *negotiating* (through joint assessment of convergences and divergences), *synthesizing* (by co-developing shared understandings while maintaining integrity), and *applying* (as knowledge will be useful to multiple actors) knowledge (Tengö et al., 2017)	

**Table:** 

Community Based Participatory Research for health (CBPR)	
**Community Based Participatory Research** is a transformative research approach that bridges the gap between science and practice in order to eliminate health inequities (e.g., due to racial, ethnic, ability, socio-economic and gender disparities) as well as to improve the health and quality of life of communities (Israel et al., 2013). CBPR enhances problem understanding (included needs, barriers, and assets) and integrates the knowledge gained for the co-design and implementation of interventions and policy for social change. CBPR equitably engages communities as co-creators (e.g., community members, organization representatives) in all aspects of the research process (from design to implementation and evaluation) (Wallerstein & Duran, 2010). CBPR has been applied to numerous participatory and collaborative projects and programs, in relation to multiple health issues (e.g., preventive medicine and sexually transmitted diseases) and a wide variety of communities (e.g., women in difficult situations and sexual or gender minorities) (Frahsa et al., 2011; Rhodes et al., 2021a) (see Sect. 7) ***Collaboration and co-creation*:** CBPR proposes that *context* (e.g., social determinants of health or historical legacies of injustice) grounds *collaborative dynamics* between researchers and communities (e.g., from structural to individual and relational), which can impact and change the *design* of research and interventions contributing to specific *outcomes* (e.g., improved community capacities and health outcomes) (Belone et al., 2016)**.** Central to the collaboration process is the cultivation of relationships through: trust-development (as a commitment to building trusting relationships sustain collaboration over time), mutual learning (as bi-directional translation, implementation and dissemination ensure relevance and usefulness), and consideration of power dynamics (to redress power imbalances and address health inequalities) (Freudenberg & Tsui, 2014; Jagosh et al., 2015) ***Evidencing process*:** CBPR questions understandings of evidence in health research that focus exclusively on methods largely developed in academic settings (e.g., RCT). Essential to CBPR is that community partners have a voice and that their cultural norms, values, and knowledge inform research design and implementation through mutual learning (Wallerstein & Duran, 2010). Validation of information (e.g., biophysical data, descriptions of socio-cultural contexts, interpretations of outcomes) for the co-creation of evidence can be achieved through multiple collaborative methods (e.g., member checking, reflection activities, respondent validation, assessment of co-creators’ engagement and enjoyment) (Leask et al., 2019). Realist evaluation methodology is often used to assess CBPR as it foregrounds questions such as: What works? For whom? Under what circumstances? Why? And how? Realist methodologies starts from explicating the underlying assumptions about the functioning of complex mechanisms (through logic models of planned interventions); co-creates research questions and data collection protocols; and uses range of quantitative and qualitative data towards the refinement of the mechanisms (Jagosh et al., 2015)	

## Embracing the complexity of mixed mechanisms in co-production

In philosophy of medicine and in medical methodology, scholars have discussed extensively the role and meaning of evidence for establishing medical knowledge and for designing interventions. An important result of this body of literature is the attention to evidence of mechanisms, alongside evidence of correlation as is generated in quantitative studies and especially in randomized controlled trials and meta-analyses. We adopt here a ‘minimal’ definition of mechanism, according to which “a mechanism for a phenomenon consists of entities (or parts) whose activities and interactions are organized so as to be responsible for the phenomenon” (Glennan et al., 2022, p. 145). In the context of complex health or environmental challenges, the idea of mixed mechanisms may refer broadly to how both human or environmental health depends on social and political factors as well as on bio-chemical (e.g., in response to drugs or various types of exposures) or bio-physical ones (e.g., CO_2_ emissions). Indeed, mechanisms involved in human and environmental health are at least bio-social, where ‘social’ refers to a whole range of factors from socio-economic to cultural or psychological ones, and in this sense these mechanisms have been dubbed ‘mixed’ (Kelly & Russo, 2018; Kelly et al., 2014).

Mixed mechanisms are not machines or ‘machine-like’, engineered objects, in which it is easy or even possible to identify a single factor to bring about change in another factor (Ghiara & Russo, 2019; Kelly & Russo, 2018, 2021). Rather, they represent a way to capture the complexity of the situations in which decision-making and action take place while accounting for the numerous factors and dynamics that play a role, such as biological, social, cultural, economic and technical. These different kinds of factors interact with one another, and at different scales: the scale of individual action, of group action, of social pressure, of institutions. We insist on the idea of a mixed *mechanism*, because mechanisms carry explanatory power. That is, we are forced to give *some* description of the working of the mechanism, of the factors and actors thereby involved. In the language of the philosophy of mechanisms, the *activities* and *organization* of the mechanism are key (Glennan & Illari, 2018). But it is important to note that mixed mechanisms carry explanatory force only because different *actors* as epistemic agents in the participatory process do the intervening as well as the explaining. These epistemic agents  do not just develop descriptions of such mixed mechanism, but they also interact with each other and with the context in which they operate.

Working within and through mixed mechanisms, participatory and collaborative approaches move beyond more conventional ways of doing science and engage in situations characterized by normative uncertainties and political conflicts, such as working in highly disadvantaged groups, or dealing with the conflicting interests of the local population and of the government (Chambers et al., 2021; Freudenberg & Tsui, 2014; Turnhout et al., 2020; Valles, 2018). Thinking in terms of mixed mechanisms invites getting clear about the multiplicity of factors that enter decision-making and action processes, such as socio-economic-cultural factors. Further, mixed mechanisms invite thinking about the broader dynamics that include researchers as part of the problem they intend to study. Qualitative and participatory approaches in social science have been often criticized for introducing an irreducible element of subjectivity and bias in the research (Montuschi, 2017). However, this kind of criticism does not consider how participation, when accompanied with appropriate reflexive practices, generates more appropriate and relevant evidence than the alleged objective method typical of evidence-based approaches (Minkler et al., 2010; Wallerstein & Duran, 2010). Moreover, qualitative methods have undergone significant developments, for instance through data synthesis and techniques like meta-ethnography, which have greatly improved the reliability of sampling and results (Dixon-Woods et al., 2005).

## The *how *and the *who* in participatory and collaborative research

In participatory and collaborative approaches in sustainability science and public health research different actors work together (the who) to support decision-making processes and interventions (the how) to address complex problems in mixed mechanisms. Thus, in other words, participatory and collaborative research in mixed mechanisms forces us to reflect on how evidence emerges from and is utilized in relation to deliberation and action processes (the how) designed and implemented collaboratively by multiple actors (the who).

### The how: Action-orientation for design and implementation in context

Participatory and collaborative research is embedded in action processes that allow for designing and implementing interventions and measures collaboratively in specific contexts (Caniglia et al. 2021). The role of *context* in designing, enacting and implementing interventions is essential, as what works in one context might not work in another one (Cartwright & Hardie, 2012; Dobrow et al., 2004). Further, context matters when considering the knowledge and evidence that can be generated and includes “all factors within an environment where a decision is made” characterized by its complexities, comprising knowns and unknowns as well as certainties and uncertainties (Dobrow et al., 2004). Some aspects of the contexts can be influenced and manipulated and some others are outside of the reach of an intervention (Caniglia et al., 2017), such as disease-specific aspects in public health or extra-jurisdictional and political factors in sustainability science. Context cannot be relegated to background conditions or to supportive factors, but are an integral part of decisions and actions (Montuschi, 2017). It is thus of utmost importance to embed considerations about contextual factors and how they enter decision-making and action in complex mixed mechanism.

### The who: Actors-orientation through mutual learning

By definition, participatory research actively involves different actors not as passive objects of investigation but as active epistemic agents and emphasizes collective inquiry and experimentation grounded in their experiences as well as in their personal and social histories (Chevalier & Buckles, 2013). Participatory formats aspire to mobilize the diversity of values and experiences of multiple actors, especially those most affected by a problem. Their personal experiences, histories, values and worldviews are brought into the research process, where knowledge generation equates with action as well as with mutual learning and capacity building (Caniglia et al., 2021; West et al., 2019). A major issue emerging in participatory research has to do with the differential capacities of the epistemic agents involved (Knickel et al., 2023; Avelino, 2017; Turnhout et al., 2020). Power relationships and asymmetries can determine whether or not collective learning processes are fostered and how knowledge and evidence are generated (Fritz & Meinherz, 2020). There is increasing recognition that unless the process of collaboration is reflexive and well- structured, intentions to empower often end in dynamics of *dis*empowerment and can become an obstacle to generating both environmental and health justice (Caniglia et al., 2023). Often, evidence from scientific studies is even used in questionable ways to silence minorities’ voices and increase disparities of access to resources (Turnhout et al., 2020; Turnhout et al., 2010). It is thus important to elaborate on ways of dealing with evidence that help to minimize these dynamics of disempowerment.

### The how and the who: evidencing through co-production in mixed mechanisms

The co-production of *evidence for action* is inherently mixed, across disciplines, epistemologies and multiple values and knowledge systems (Johnson et al., 2017; Tengö et al., 2014). Participatory and collaborative research that takes place in mixed mechanisms thus requires highly hybrid methodologies to be investigated and intervened upon, such as methods from the natural sciences (e.g., meta-studies or RCTs) or from the social sciences (e.g., surveys or ethnographic research). Furthermore, evidence is also gathered through transdisciplinary methodologies that include non-academic knowledge systems (Fazey et al., 2018; Lang et al., 2012). The generation of evidence in participatory and collaborative approaches requires learning-based forms of evaluation (Knickel et al., 2023; Luederitz et al., 2017). Evidence for action needs to account for the multiplicity of kinds of knowledge and ways of knowing, such as citizens’, policy makers’ and administrators’ knowledge, depending on the context and issues addressed (the mixed mechanisms alluded to earlier). They thus account for and combine multiple standards of knowledge and evidence generation and utilization.

## Clues from Susan Haack: evidence as *clues for action*

Considering the kind of participatory and collaborative research that supports the generation of evidence for action in mixed mechanisms, we need more nuanced and fitting perspectives to understand what we mean by evidence in this kind of research. In order to start elaborating such a perspective, we build on philosopher Susan Haack’s theory of evidence, which has been highly influential in legal thinking and applied sciences but less in epistemology and philosophy of science (Haack, 2001, 2011, 2014). Haack’s work in epistemology and theory of knowledge combines two opposing stances on the role of evidence in the justification of knowledge claims: *foundationalism* (that is, the ultimate justification for knowledge does not depend on other beliefs but only on how knowledge is well anchored empirically) and *coherentism* (that is, a belief, or set of beliefs, is justified in case it is part of a coherent set of beliefs, from a logical point of view) (Haack, 2001, 2014)**.** Haack uses the term *foundherentism* to define her position as merging *foundationalism* and *coherentism*: like (some forms of) foundationalism, Haack’s foundherentism allows a role for empirical data generated; like (some forms of) coherentism, it allows for pervasive relations of mutual support (or of contradiction) among beliefs (Lagier, 2020). It is by balancing foundationalist and coherentist stances that evidence for a claim can be generated.

We are aware that both foundationalism and coherentism are the object of a long-standing and vivid debate in epistemology (Olsson, 2021). Without privileging one of the two, we think that a major strength of Haack's theory of evidence consists in claiming that either approach, on its own, is incapable of providing an account of how our beliefs are justified and can thus have evidential role. Admittedly, Haack's foundherentism is not a full-blown account of evidence, and remains rather evocative; it is beyond the scope of this paper to provide such an account, but we think that’methodological pluralism’ and the ‘correctness theory of truth’ can give us the tools to spell out foundherentism (Floridi, 2013; Russo, 2022). Briefly put, this would mean to retain the main idea behind coherentism, but without giving ‘coherence’ a strict, logical sense. In fact, in numerous research contexts, coherence cannot be reduced to a mere property of any set of propositions or formal model. Instead, coherence’ should be given a broader meaning that embraces the *whole* process of knowledge generation and validation, from hypothesis generation to interpretation of results; this ‘holistic’ view also allows for a pluralistic approach that is appropriate to account for evidencing practices in MEB and CBPR. Methodological pluralism would then need to be coupled with the correctness theory of truth, abandoning any narrow view of truth in terms of correspondence between well-formed propositions (in natural or formal language) and the world. In the correctness theory of truth, the truth of scientific claims is established *within* a given framework, which embeds the whole of methodological choices made by researchers as well as values and norms. Following Haack on her definition of foundherentism we can thus retain elements related to coherence (but cashed out as coherence of the whole modelling process) and also about foundations (i.e. what the world is like and what we can claim about).

Without the aspiration to exhaust the richness of Haack’s work on evidence, we suggest that Haack’s foundherentism can provide adequate directions to think about *evidence for action* in sustainability science and public health. Haack defines *evidence as clues* which emerge from “… a mesh of many threads of varying strengths anchored more or less firmly in experience [foundationalism] and woven more or less tightly into an explanatory picture [coherentism]” (Hack 2001, p. 254). Although Haack’s notion of evidence has been mainly used in legal contexts and legal reasoning, she suggests that it may be possible to use it also in other research contexts as: “The evidence with respect to scientific claims is like empirical evidence generally–only more so: more complex, more dependent on instruments, and usually a shared resource” (Haack, 2001, p. 253).

### Evidence as clues in a crossword puzzles: The evidencing process

Haack makes use of the crossword puzzle analogy to explain in simple terms how empirical insights, such as quantitative and qualitative data, on the one hand, and a multiplicity of beliefs, on the other hand, contribute to generating evidence (Haack, 2001, 2014). According to Haack: “The evidence with respect to factual, empirical claims is a complex mesh in which experiential evidence, i.e., the evidence of the senses, and reasons, i.e. background beliefs, work together like the clues and ramifying intersecting entries in a crossword puzzle” (Haack, 2014, p. 30). Indeed, data alone are not evidence for or about a claim until epistemic agents think about *how data are generated* (the foundherentist aspect of when we ask, for instance: Are the data reliable or not?) or *how we can make sense of data* in relation to other beliefs (the coherentist aspect of when we ask: Is there coherence or contradiction between different beliefs?). From this perspective, evidence generation is similar to filling a crossword, in which one tries to appropriately intersect words including a person’s background beliefs ramifying in different directions (coherentism), while giving answers to clues that we gather from our experience and knowledge of the world (foundherentism). Clues come from the methods used, as well as from the positioning and situatedness of the researcher (or group of researchers). Most importantly, clues come also from a variety of methods used, including those of ‘clinching’ or ‘vouching’ evidence, to use an expression from (Cartwright and Efstathiou 2009). This means allowing for methods that aim at studying very specific aspects of causal relations (e.g., RCTs) as well as methods that study problems and phenomena from a broader perspective, as for instance MEB and CBPR do.

The simplicity of the crossword analogy may seem to be at odds with the complexity of participatory and collaborative research. Yet, we think that Haack’s theory of evidence as clues, with the analogy of the crossword puzzle, can help to capture important but often-overlooked features of the evidencing processes in knowledge co-production, which we do not see if we establish a priori hierarchies according to methodological considerations. First, the theory invites reflecting on the many kinds of insights that may count as clues for action (such as insights gathered through participatory observation or from interviews with local actors; or quantitative data gathered from epidemiological studies). Second, the crossword puzzle analogy expresses the need to consider how clues are mobilized within existing entries, that is in relation to existing systems of beliefs, values and worldviews and never in a vacuum. And this in ramifying and not linear ways, similar to what happens when one starts looking for multiple clues when filling a crossword puzzle. Third, conceiving of evidence as clues does not aim to get at complex mixed mechanisms as if they were entities to be picked like cherries on a tree or as allowing for cracking the code and opening the box to see exactly how things work. So, to return to Cartwright’s influential distinction, clues do not clinch evidence (but do not vouch it either). Rather, considering evidence as clues for action invites the co-construction of knowledge in relation to the many bio-social-ecological and technological workings of mixed mechanisms. Adopting the idea of ‘clues’ allows us to consider an element that traditional epistemological accounts of evidence (or of causality, as in the case of Cartwright) do not consider, namely the role of epistemic agents, or actors, in co-constructing knowledge. This is especially important in participatory and collaborative research where it is central to consider that it is not (just or only) a question of which methods will deliver the best evidence, but primarily and foremost a question about how knowledge emerges from processes of co-production.

### Evidence and the role of actors as epistemic agents

Beside the crossword analogy, Haack’s theory also allows for focusing on *actors*-oriented dimensions of evidence generation and mobilization. Haack writes: “Warranted scientific claims are always warranted by somebody’s, or somebodies’, experience, and somebody’s or somebodies’; so a theory of warrant must begin with the personal and then move to the social before it can get to grips with the impersonal sense in which we speak of a well-warranted claim or ill-founded conjecture” (Haack, 2001 , p. 253). This quote points out the need to think about evidence in relation to the individuals or to the collective actors who generate, mobilize or utilize evidence, where actors refer to any epistemic agents involved in the process. It also shows that any attempt to make evidence objective via methods *only* and in abstraction of *who* handles such methods will return that is partial and perhaps even wrong.

Existing accounts in philosophy of science and in medical methodology often do not consider explicitly the role of different epistemic agents in the process of evidence generation. Evidence in participatory and collaborative settings is instead inherently actor-oriented, as there are always actors involved contributing to generating and selecting the clues within the complex workings of mixed mechanisms. The role of epistemic agents has been neglected in philosophy of science debates, and long associated with social construction in social studies of science; our move, instead is to include explicitly actors in this picture, for their epistemic role, not just their social role (Russo, 2022). Haack’s account invites thinking about the multiple (and not always aligned and coherent perspectives) that different actors might bring in determining what counts as evidence and whose evidence counts in participatory processes (Ludwig & El-Hani, 2020; Tengö et al., 2014). Here, it is through the agency and empowerment of people that decisions are taken and actions or programs implemented. Haack’s theory of evidence can help us to bring the actors as epistemic agents with their positionalities and standpoints into consideration when considering what we mean by evidence in participatory and collaborative research. In participatory contexts, it might be neither possible nor desirable to aim for total coherence and fit. Rather, opening this space for confrontation across multiple perspectives allows for considering the many trade-offs and tensions that present themselves fostering processes of mutual learning across different constituencies (Knickel et al., 2023; Hirsch & Brosius, 2013).

## Procedural criteria for the co-production of *evidence as clues*

Haack distinguishes between *criteria for justifying beliefs* and *procedural criteria* for the conduct of inquiry. The former are like the criteria for judging whether or not a meal is nutritious and the latter are like instructions for cooking it (Lagier, 2020). The procedural criteria are both descriptive and normative. As descriptive, they allow for making sense of specific aspects of participatory and collaborative research. As normative, they may be used as guidelines helping to determine the strategy that should be followed to carry out a good investigation. They help to spell out the complex trade-offs used in the implementation and evaluation of research through different lenses (i.e., valuation, governance, power, scientific evidence, methods) (Russo & Hirsch, 2023). In this section, we present the general characteristics of the six procedural criteria, inspired by Haack’s account (Haack, 2001). In detailing each criterion, we expand Haack’s original formulation through other works from philosophy of science, social epistemology, social studies of science and political sciences. In Sect. 7, we then detail how the procedural criteria may help to make sense (as descriptive) and to guide (as normative) research in specific examples of the MEB (Box 1) in sustainability science and of CBPR (Box 2) in public health.

### *Evidence as action-oriented clues *(the *how*)

Criteria related to the *how* can provide guidance on the evidencing practices that allow for generating new evidence in and through action as well as on how to make use of existing evidence when making decisions and taking action through participatory research.

*(a) Foundherentist:* Balance considerations about where clues come from (foundationalism) and about how they may relate to different perspectives (coherentism).

As highlighted above, Haack’s epistemological perspective combines foundationalist and coherentist considerations through the notion of foundherentism. From a foundherentist stance, first, the value of evidence as clues should be assessed looking at how those clues emerge from empirical research, such as when asking: How are clues generated? Who is bringing them about? How can we assess them? This is the foundational dimension. These questions, for example, are asked when Indigenous or local knowledge are validated with their own knowledge systems in the MEB, such as the knowledge of local farmers about the pollinator crisis or of Indigenous communities about environmental risks in their own land (see Sect. 7.1). Second, the role of clues should be evaluated in relation to the different knowledge systems involved by asking, for example: Is there coherence? Or partial overlap? Or incompatibility? (Ludwig & El-Hani, 2020). This is the coherentist dimension. Such considerations span ethical, epistemological, and ontological considerations when comparing, bridging, and synthesizing insights from different (and internally-validated) knowledge systems in the MEB; or when discussing in CBPR differences and similarities between perceptions and experiences of community members in relation to existing scientific literature, as exemplified in Sect. 7.

*(b) Gradational:* Critically evaluate the criteria used to assess the strength of different kinds of clues as evidence for action.

Haack considers evidence as gradational, in the sense that it is not categorical, as responding to yes or no questions, but rather a matter of degree as evidence with respect to a claim may be stronger, or weaker (Haack, 2014). Acknowledging the gradational nature of evidence invites us to think about how we determine what makes some clues stronger or weaker than others, not in absolute terms, but depending on the problem addressed, on who is affected by it, and on the complexities of the mixed-mechanisms in which research takes place. A gradational approach makes clear that knowledge uncertainty cannot be eliminated and that proposed ‘principles of total evidence’ or strict evidence hierarchies ultimately remain unrealistic (Good, 1966). The MEB and CBPR, for example, acknowledge the central role of power imbalances and historical legacies of injustice in determining the strength of evidence. Both approaches are motivated by the need to counterbalance the effect of such imbalances and legacies when working across multiple methodologies (e.g., when including qualitative research in systematic reviews) and epistemologies (e.g., in the synthesis of insights from different knowledge systems). As exemplified in Sect. 7, in these research contexts, a gradational understanding of evidence invites asking, for example: What factors (e.g., power imbalances and historical legacies) contribute to determining the strength of evidential support of a study? What kind of evidence do quantitative data (or qualitative) provide to the understanding of complex mixed mechanisms? What knowledge gains are offered by control-based design, and are they necessary to guide action?

*(c) Quasi-holistic:* As multiple perspectives and methods provide different clues for action within complex mixed mechanisms, develop integrative ways to deal with them.

Haack’s theory of evidence straddles a middle ground between atomism (focusing on one claim or one situation at the time in isolation) and holism (where the full complexity of a situation is kept into consideration) as “The evidence relevant to a claim is usually complex and ramifying; but not everything is important to everything” (Haack, 2014, p. 15). Quasi-holism speaks to the importance of having comprehensive perspectives, which include multiple factors and perspectives when dealing with complex mixed mechanisms (holism) while also selecting most relevant factors and aspects (quasi). Both the MEB and CBPR emphasize the importance to include multiple perspectives when addressing complex situations (holism), but also prioritize the need to give voice, mobilize and leverage the often-neglected perspectives of marginalized and vulnerable actors as they are disproportionally affected by health and environmental threats (quasi). The examples in Sect. 7 show how quasi-holism invites moving away from questions framed in terms of “How much evidence do we have?” towards questions such as: How do we set priorities when selecting among different sources of evidence (e.g., qualitative or quantitative) in mixed methods and mixed data approaches and research design? But also: Are priorities set up through scientific criteria, arguments, and standards enough and most appropriate? How do these priorities determine perceived limitations and strengths or any piece of information we might have? How do we decide what clues are relevant and what not?

### *Evidence as actors-oriented clues *(*the who*)

Criteria related to the who provide guidance on the epistemic practices that allow for generating evidence in and through action as permeable to socio-political (such as power), personal (such as perceived needs and worldviews), and experiential factors (such as tacit knowledge).

*(d) Social:* Consider that evidence generation and use are permeable to socio-political factors and dynamics.

Haack provides an account of warrant that highlights the interpersonal and social dimensions of evidence generation in collaborative settings, such as when relying on others’ testimony, knowledge and expertise (Haack, 2011). Indeed, deep power differentials and historical legacies of injustice contribute to prioritize what are considered effective practices and solutions towards health and sustainability (Echo-Hawk, 2011; Tengö et al., 2014). Power and interpersonal dynamics are not external to the generation and evaluation of evidence (Caniglia et al., 2023; Fricker, 2007). Rather, they influence what is considered acceptable evidence, what evidence is available, and what role evidence may play in informing policies or in re-directing decisions (Avelino, 2017; Freudenberg & Tsui, 2014). MEB and CBPR, for example, explicitly “give voice to communities” that have been historically discriminated or disadvantaged having them participate in the whole research process as detailed through the different examples in Sect. 7. Central questions that foreground issues of epistemic and social justice in knowledge co-production are for example: Who makes decisions and whose evidence counts? What power structures and inequalities underly these judgements? Who is framing the problem and shaping the processes? Who is given voice in these processes and who is silenced? Who sets up the research, including the background of the research? Who evaluates the evidence?

*(e) Personal:* Consider that personal motivations and interests as well as the positionality of different actors influence what evidence is generated or how existing evidence is used.

Beside the social dimensions, Haack focuses on the evidence that actually leads someone to believe something at a given time (Haack, 2014). She proposes an account of justification that is *personal* because it depends on the quality of the evidence that leads a person to have a certain belief, to believe in something. The personal and situated character of knowledge, and by extension of evidence, is also a topos of feminist approaches and it has to be considered for its epistemic significance too (Haraway, 2020). As exemplified in Sect. 7, both CBPR and MEB mobilize and leverage the personal experiences and perceptions of those affected by health and environmental problems, such as when designing programs to reduce transmission of sexually transmitted infections among LGBTQI + people (Rhodes et al., 2021b) or when mapping the value local people attribute to specific waterscapes or fauna when deliberating about ecosystem governance (Robinson et al., 2015). Questions to be asked in the evidencing process may be, for example: How can we consider personal considerations not *ipso facto* biases of otherwise objective/neutral stances? How can we leverage the insights of actors speaking from certain positionalities, because of race, ethnicity, gender, sexuality as well as on wealth or social-economic status? How can we assess that researchers are tied to certain ideas about how data should be evaluated, collected, and interpreted in decision-making processes?

*(f) Embedded:* Look for clues not only in the form of explicit knowledge, but also in tacit, embedded and embodied forms of knowledge.

Haack’s theory of evidence takes the evidence with respect to empirical claims to include forms of knowledge, that are often tacit and not expressed in propositional language (Hack, 2014). Knowledge relevant for decisions and action for sustainability and health is often embedded, for example, in the rituals and traditions of local communities (Fazey et al., 2014; Tengö et al., 2014) or in their social networks, such as mutual-aid informal networks. Acknowledging the importance of embedded forms of knowledge is especially important when some actors may not have the same capacity to generate propositional knowledge as others, because of language differences, education, or differences in culture. In Sect. 7, we show how both MEB and CBPR make use of methods that allow for capturing expertise and tacit knowledge, such as through mental maps or photovoice, and structure the collaborative process in ways that allow for eliciting and mobilizing embedded and tacit forms of knowledge (Fazey et al., 2018; Israel et al., 2013). Questions underpinning these processes are, for example: What might be knowledge and insights that are usual research approaches do not allow us to see? How can be find rigorous and creative ways to bring them to expression? What methods are least invasive and most appropriate for this?

## Examples of collaborative and participatory evidencing practices

In this section, we show how the six procedural criteria can help to account for evidencing processes in studies that have used the MEB approach in sustainability science (Sect. 7.1) and CBPR in public health research (Sect. 7.2). We detail how in the collaboration and co-creation as well as in the evidencing processes, the six procedural criteria highlight complementary, though often also overlapping, aspects of participatory and collaborative research dealing with research about: declining pollinator diversity and food security in India (Smith et al., 2017), environmental risk assessment with Indigenous people in New Zealand (Robinson et al., 2015) as well as health prevention for women in difficult life situations in Germany (Frahsa et al., 2011) and reduction of sexually transmitted infections in LGBTQI + communities in the USA (Mann-Jackson et al., 2021).

### Assessing validity through the Multiple Evidence Base approach﻿

#### Declining pollinator diversity and food security (India)

An example of evidence validation using the MEB is a study aiming to address the problem of declining pollinator diversity and abundance in the Orissa region (India) (Smith et al., 2017). Pollinators’ decline affects food security for subsistence farmers who meet large part of their nutritional needs from a variety of pollinator dependent vegetable crops. In the Defra Darwin Initiative, it became clear that there was a lack of knowledge about: the diversity of the crops grown and trends of productivity in the study area, pollinators’ identity and trends in abundance and diversity, what pollinators are important for crop pollination, and whether changes in crop productivity are linked to changes in pollinator diversity and abundance.

*Collaboration and co-creation:* In order to fill the gaps above and determine evidence-based management strategies that would fit the social context, researchers engaged with farmers and their experiences of the local environments, crops, and pollinators (e: personal). Following the main assumption of the MEB, that is the need for finding validation mechanisms that are internal to different knowledge systems, a system of peer-to-peer consensual validation was established (f: embedded). Through a series of workshops, peer groups of farmers (instead of scientific experts) validated each other’s knowledge as experts holding the same or similar values, worldviews and mental models (d: social).

*Evidencing process:* First, questionnaires were co-created by researchers and farmers in order to gather evidence about facts (e.g., How many pollinators are present in the field?) and inferences about potential explanations of those facts (e.g., Do pesticide affect pollinators?) (a: foundationalist side of foundherentism). Local farmers were asked in groups to verify the sources of their knowledge (e.g., How do you know that pesticide affect pollinators?) and to elaborate on solutions (e.g., Would it be useful to have more pollinators? How could their abundance be increased?) (a: foundationalist side of foundherentism). Afterward, farmers from a different group were asked to review other groups’ statements in discussion groups were, first, statements were read out loud and, then, farmers could accept, reject or modify other groups’ statements (a: coherentist side of foundherentism). The evidence generated in this way provided relevant perspectives on local crop yields and pollinator trends in Orissa **(**c: quasi-holistic). The evidence generated allowed for further interrogating the problem (e.g., when discussing how to better determine how the number of pesticide influences pollinators decline) and for assessing solution options (e.g., how to restore pollinator populations by reducing pesticide use, restoring natural habitats, and introducing bee boxes). The continuous assessment of the evidence also involved other sources, e.g. from other locations and scientific studies (b: gradational).

### Environmental risk assessment in Indigenous knowledge (Australia)

In a second example, the MEB approach was used in the Girringun territories (Northern Australia) to conduct an assessment of risks due to rapid environmental and climate change, which affected efforts to sustain resilient landscapes and threatened the livelihoods of local, Indigenous communities (Robinson et al., 2015). Environmental and climate change affected local watershed, reducing access to and quality of water in local rivers and streams, and local fauna as exotic pests impacted native plants and trees. They also negatively impacted the health of community members (included mental health and spiritual well-being) as well as the quality of food and plant resources with which they supplement their daily diet, especially given their low average income. The project thus aimed at facilitating knowledge sharing between indigenous people about the assessment of environmental risks in their territories.

*Collaboration and co-creation:* Also because of the lack of trust towards researchers and their science-based planning solutions, an iterative and collaborative research design was implemented, based on a transparent agreement on intellectual property rules, clear roles and responsibilities of participants, and resources for involvement (d: social). All participants co-developed criteria and standards for the evaluation of the research partnership. Indigenous people, especially the Elders, were engaged in the co-design of the project from the outset. They suggested appropriate thematic focus on watershed and pests as major issues for the communities. They also made decisions on locations and design of workshops and who should be invited (e: personal; f: embedded). Evaluation of the quality of the knowledge partnerships was foregrounded throughout the project (d: social).

*Evidencing process:* Researchers suggested the use of participatory mapping, in order to capture local communities’ values, perspectives, and needs that could not be easily verbalized (embedded). These methods allowed for diversity among the knowledge claims of different Indigenous communities to be acknowledged. Participatory mapping followed several steps. First, it entailed the creation of individual maps (depiction of rivers, values attached to the rivers, and the partnerships that allow for sustaining these values) (a: foundationalist side of foundherentism). In these maps, participants highlighted how and why they valued freshwater places, species and cultural resources because they provide food, shade, and habitats for important plants and animals (personal). Further, each group generated collective maps (a: coherentist side of foundherentism). The subsequent discussions of the collective maps across groups highlighted the diversity of indigenous knowledge systems in the region (e.g., different families and clans) and the multiplicity of values attributed to the affected environment (c: quasi-holistic)**.** Discussions around the maps emphasized how Girringun’s legal and knowledge systems have had to find new ways to accommodate social environmental and cultural pressures in their ancestral lands (d: social). The collective assessment of environmental risks and knowledge partnerships validated by participants were cross-fertilized with scientific understandings of risk assessment and effective knowledge partnerships (b: gradational).

### Evidence co-production and community based participatory research

#### Health prevention for women in difficult life situations (Germany)

In a first example, CBPR was used to develop, implement and evaluate sport programs for physical activity working with women in “difficult life situations” in the city of Erlangen (Germany) (Frahsa et al., 2011, 2012; Rütten et al., 2023). Women with low income or educational attainment, unskilled occupations or belonging to ethnic minorities are among the least physically active, with high prevalence of sedentary life-styles, and thus high levels of associated conditions, such as obesity or cardiovascular diseases. Yet, few studies provide concrete advice on how to plan and implement health-promoting projects and even fewer focus on movement through participation. The *BIG project* studied how to make full use of potential effects of physical activity in health promotion going beyond a biomedical focus on health and foregrounding psychosocial, educational, and environmental dimensions of health. This implied developing evaluation instruments for health promotion adequate to the people and contexts involved.

*Collaboration and co-creation:* Instead of aiming at behavior change through ready-made interventions, BIG established a co-operative planning group including researchers, women from the neighborhood as well as decision-makers, and local experts (d: social). The group made decisions together on planning, implementation, and evaluation of activities (e.g., low fee exercise classes with childcare, accessible sport facilities, education activities, and training for improved organizational capacities). Different stakeholders provided different resources (e.g., sport facilities by sport associations, funding by policy makers). The collaboration foregrounded the needs and priorities of the women (e.g., by incorporating low-threshold interventions) (e: personal; f: embedded).

*Evidencing process:* BIG opted for a mix-methods approach to generate evidence about reach, implementation, and maintenance of health promotion activities (c: quasi-holistic). Quantitative methods were used, for example, to measure heart-rate variability as indicator of psychosocial stress (a: foundationalist side of foundherentism). Qualitative methods were used to assess benefits at the social organizational and policy level (e.g., through focus groups and individual interviews) (a: foundationalist side of foundherentism). All were used for output assessment (implementation) and process evaluation (planning) through focus groups, interviews, policy ethnographies (a: coherentist side of foundherentism). The central results of BIG were outside of evidence-based medicine paradigm. In the co-design of studies and interventions, for example, women opposed the idea of randomization and of a control group that would have not benefitted from the program and demanded that all women should get the chance to immediately participate (d: social). This choice determined the kind of evidence that could be gathered as well as the level of participation and empower of community participants (d: social). Women reported increased social networks and beneficial effects as well as increased self-efficacy, felt empowered to voice their interests (e: personal).

### *Reducing sexually transmitted infections in LGBTQI* + *communities (USA)*

In another example, CBPR was used to address the disproportionally high rates of Sexually Transmitted Infections (STIs) and HIV among LGBTQI + people (especially men who have sex with men and transgender women of color) in North Carolina (United States) (Rhodes et al., 2012; 2021a). A series of studies aimed to identify needs, priorities and assets related to STI/HIV prevention, screening and treatment as well as to social determinants of health among LGBTQI + in North Carolina (Mann-Jackson et al., 2021). Partners used the findings to inform the development of innovative, multilevel, and meaningful interventions to reduce STIs and HIV infections and improve social determinants of health in the communities.

*Collaboration and co-creation:* The series of studies and projects was conducted by a long-standing CBPR partnership that comprised public health researchers, LGBTQI + community members, and representatives of community organizations, who are rarely engaged through authentic power-sharing approaches (d: social**)**. The partnership worked collaboratively with a Community Advisory Board. In the series of studies mentioned above, the CBPR process engaged first community members identified as informal leaders and with representatives from health organizations (e.g., in community clinics and HIV-serving organizations) (e: personal) or organizations dealing with social determinants of health (e.g., job training programs, community foundations, and immigrant-serving organizations). The partnership then expanded to more participants identified and nominated by community members (f: embedded).

*Evidencing process:* The first part of the study was about generating evidence about the needs, priorities, and assets of the community (personal, embedded). Numerous interviews allowed for exploring experiences and perceptions related to sexual and general health and social determinants (health, employment, education, social support, discrimination) (a: foundationalist side of foundherentism). Aiming to capture a wide array of experiences through constant comparison, CBPR partnership members read and reread interview notes, compared and contrasted content categories based on each member’s interpretation of the data, and identified emerging themes (a: coherentist side of foundherentism). In the second part of the study, CBPR partnership members revised and re-interpreted themes to develop intervention strategies. First, preliminary themes were presented and discussed to interpret findings (e.g., What do you see in these interview data?) and assess priorities (e.g., Which of these findings are a priority for making a difference in the lives of those most affected?) (b: gradational). Then, an iterative health promotion planning process was used to develop intervention strategies that could address community needs and priorities and leverage community assets identified in the needs assessment (c: quasi-holistic). Three primary strategies were designed (a community-based peer navigation; use of social media; and anti-discrimination trainings for organization staff) and integrated into the bilingual Impact Triad intervention, whose implementation and evaluation are still ongoing.

## Clues and procedural criteria for practical objectivity

The application of the six procedural criteria to studies using MEB and CBPR shows how evidencing processes take place in forms of inquiry that work in integrated ways with multiple kinds of knowledge and ways of knowing (e.g., from different actors or studies) (Russo, 2022; Tengö et al., 2014). By combining action- and actor-oriented considerations, the examples above make clear that there are no methods for the generation of evidence that are intrinsically better or worse than others (as postulated in the usual evidence hierarchies). Rather, thinking about evidence as clues for action invites thinking of typologies of evidence where different methods might provide different insights about decisions to take and interventions to pursue (Petticrew & Roberts, 2003).

Overall, thinking about evidence as clues and as always connected to the activities, practices, and lives of epistemic agents (the how and the who) is a reminder of the *fragility* of evidence for decisions and actions. Indeed, evidence does not provide certainty, but rather like the clues in a crossword puzzle, it can support decision-making and actions by allowing for filling in some of the information; may need to be revised constantly; and may require further inquiry in the attempt of justifying why certain decisions should be taken in specific moments and situations. The procedural aspect of the criteria marks an important difference with existing evidence-based approaches in that there is no attempt to put pieces of evidence into rigid boxes, let alone into rigid hierarchies. Interpreting evidencing processes through the six procedural criteria rather shows that the integration of different insights cannot be done through strict and rigid protocols, for instance for the production of meta-analyses or following action-protocols (Barrotta & Montuschi, 2018). Instead, the generation and evaluation of evidence as clues for action can take place through (ideally) pluralistic, democratic, and reflexive mutual learning processes (Pohl et al., 2021)**.**

Our account of evidence as clues for action differs from philosophical accounts in relation to policies and interventions that have mainly cashed out *evidence for use* (Cartwright & Hardie, 2012; Game et al., 2018). Evidence for use has been a major contribution in accounting for the difference in establishing evidence ‘here’ and trying to export such claims ‘elsewhere’. Yet, *evidence for action* also needs to consider more explicitly issues related to deliberation and action processes on the ground (from design to planning and implementing) and the role that actors as epistemic agents play in them (Montuschi, 2017). Our account of evidence as clues for action suggests that, by combining action- and actors-oriented procedural criteria, it is possible to go beyond the often-technical and rationalistic tendencies of so-called evidence-based policies and solutions in sustainability science and public health. Moving away from the need of certainty and control, our account makes clear that what we do when generating evidence is to formulate *clues*, which are indicative and suggestive, but cannot provide a full description of mixed-mechanisms or any kind, or do not add up to form ‘total evidence’. Moreover, in combining an action- and actor-oriented perspective (the how and the who, respectively), we point out how much these two dimensions are intertwined and how considerations about one influence the other, and vice-versa.

Using these procedural criteria when conducting participatory research may contribute to the generation of a kind of *practical objectivity*, that is “built on a balance of intervening factors and on an open dialogue among the parties” (Montuschi, 2017, 61). Clearly, anything that we might claim about the complex situations in which sustainability science and public health research is embedded and about the underlying mixed mechanisms result from *multiple* studies at times with contradictory results (Parkhurst, 2016; Petticrew & Roberts, 2003; Russo, 2022). It is only moving away from a classic understanding of objectivity and moving closer to forms of practical or procedural objectivity that we can include methods and approaches for evidence generation that have been traditionally excluded from the ‘realm of objective science’. Qualitative approaches, and participatory ones especially, can thus be integrated and valued for the essential input they provide, precisely for their attention to the actor-oriented perspective. Thus, if understood through our account, evidence for action should rely on this variety of studies *as well as* on the insights, experiences, life histories of those involved in the processes, in various ways and at various stages. Evidence as clues for action can help to articulate complexity and to spell out uncertainty when dealing with the many tradeoffs that real-world decision-making contexts present (Hirsch & Brosius, 2013). Rather than aiming to best practices or best decisions, the six criteria can help to generate clues that support decision-making and interventions in ways that are more feasible and appropriate, while also aspiring to be more inclusive and just*.*

## Conclusions

Evidence-based approaches to decision-making are often advocated for the alleged role evidence has in reducing uncertainties and give a more solid basis to decision. While scholarship in policy studies discussed the role of evidence in the context of use and application, we wished here to draw attention to the function of *evidence for action* in participatory and collaborative approaches. In this paper, we suggest that evidence should not have the primary goal to reduce complexity, but rather to help to spell it out, including the possible contradictions and inconsistencies that come in situations of uncertainty, multiple worldviews and political fractures. Though not providing certainty of effectiveness or success, our account of *evidence as clues for action* offers entry points that rely on the interdependence of action- and actors-oriented dimensions of evidence generation and use. Moving from the assumption that in collective decision-making and action processes, *how is who* and *who is how*, our account of evidence as clues for action provides an innovative tool to address complex sustainability and health challenges of our time in context-sensitive and inclusive ways.

## References

[CR1] Avelino F (2017). Power in sustainability transitions: analysing power and (Dis)empowerment in transformative change towards sustainability. Environmental Policy and Governance.

[CR2] Balazs CL, Morello-frosch R (2013). The three Rs : How community-based participatory research strengthens the rigor, relevance, and reach of science. Environmental Justice.

[CR3] Barrotta P, Montuschi E (2018). Expertise, relevance and types of knowledge expertise, relevance and types of knowledge. Social Epistemology.

[CR4] Belone, L., Lucero, J.E., Duran, B., Tafoya, G., Baker, E. A., & Chan, D. (2016). HHS public access. *26*(1), 117–135. 10.1177/1049732314557084 PMC4839192 25361792

[CR5] Buyx A, Del Savio L, Prainsack B, Völzke H (2017). Every participant is a PI. Citizen science and participatory governance in population studies. International Journal of Epidemiology.

[CR6] Cairney P, Oliver K (2017). Evidence-based policymaking is not like evidence-based medicine, so how far should you go to bridge the divide between evidence and policy?. Health Research Policy and Systems.

[CR7] Caniglia, G., Freeth, R., Luederitz, C., Leventon, J., West, S.P., John, B., Peukert, D., Lang, D.J., von Wehrden, H., Martín-López, B., & Fazey, I. (2023). Practical wisdom and virtue ethics for knowledge co-production in sustainability science. *Nature Sustainability*.

[CR8] Caniglia G, Luederitz C, von Wirth T, Fazey I, Martín-López B, Hondrila K, König A, von Wehrden H, Schäpke NA, Laubichler MD, Lang DJ (2021). A pluralistic and integrated approach to action-oriented knowledge for sustainability. Nature Sustainability.

[CR92] Caniglia G, Schäpke N, Lang DJ, Abson DJ, Luederitz C, Wiek A, Laubichler MD, Gralla F, von Wehrden H (2017). Experiments and evidence in sustainability science: A typology. Journal of Cleaner Production.

[CR9] Cartwright N, Efstathiou S (2011). Hunting causes and using them: Is there no bridge from here to there?. International Studies in the Philosophy of Science.

[CR10] Cartwright N, Hardie J (2012). Evidence-based policy: A practical guide to doing it better.

[CR11] Chambers JM, Wyborn C, Ryan ME, Reid RS, Riechers M, Serban A, Bennett NJ, Cvitanovic C, Fernández-Giménez ME, Galvin KA, Goldstein BE, Klenk NL, Tengö M, Brennan R, Cockburn JJ, Hill R, Munera C, Nel JL, Österblom H, Bednarek AT, Bennett EM, Brandeis A, Charli-Joseph L, Chatterton P, Curran K, Dumrongrojwatthana P, Paz Durán A, Fada SJ, DavidGerber J, Green JMH, Guerrero AM, Haller T, IoanaHorcea-Milcu A, Leimona B, Montana J, Rondeau R, Spierenburg M, Steyaert P, Zaehringer JG, Gruby R, Hutton J, Pickering T (2021). Six modes of co-production for sustainability. Nature Sustainability.

[CR12] Chevalier JM, Buckles DJ (2013). Participatory action research: Theory and methods for engaged inquiry.

[CR13] Dixon-Woods M, Agarwal S, Jones D, Young B, Sutton A (2005). Synthesising qualitative and quantitative evidence: a review of possible methods. Journal of Health Services Research & Policy.

[CR14] Djoudi H, Locatelli B, Vaast C, Asher K, Brockhaus M, BasnettSijapati B (2016). Beyond dichotomies: Gender and intersecting inequalities in climate change studies. Ambio.

[CR15] Dobrow MJ, Goel V, Upshur REG (2004). Evidence-based health policy: Context and utilisation. Social Science and Medicine.

[CR16] Echo-Hawk H (2011). Indigenous communities and evidence building. Journal of Psychoactive Drugs.

[CR17] Eigi-Watkin J, Koskinen I (2023). Reasoning by analogy and the Transdisciplinarian’s circle: On the problem of knowledge transfer across cases in transdisciplinary. Sustainability Science.

[CR18] Evans, A., & Potochnik, A. (2020). Theorizing participatory research. In *Ethical issues in community and patient stakeholder–engaged health research* (pp. 1–17).

[CR19] Fairchild AL, Rosner D, Colgrove J, Bayer R, Fried LP (2010). The EXODUS of public health what history can tell us about the future. American Journal of Public Health.

[CR20] Fazey I, Bunse L, Msika J, Pinke M, Preedy K, Evely AC, Lambert E, Hastings E, Morris S, Reed MS (2014). Evaluating knowledge exchange in interdisciplinary and multi-stakeholder research. Global Environmental Change.

[CR21] Fazey I, Schäpke N, Caniglia G, Patterson J, Hultman J, Van Mierlo B, Säwe F, Wiek A, Wittmayer J, Aldunce P, Al Waer H, Wyborn C (2018). Ten essentials for action-oriented and second order energy transitions, transformations and climate change research. Energy Research & Social Science.

[CR22] Floridi L (2013). The philosophy of information.

[CR23] Frahsa A, Rütten A, Abu-omar K, Wolff A (2011). Movement as Investment for Health. Integrated evaluation in participatory physical activity promotion among women in difficult life situations. Global Health Promotion.

[CR24] Frahsa A, Rütten A, Roeger U, Abu-Omar K, Schow D (2014). Enabling the powerful? Participatory action research with local policymakers and professionals for physical activity promotion with women in difficult life situations. Health Promotion International.

[CR25] Freudenberg N, Tsui E (2014). Evidence, power, and policy change in community-based participatory research. American Journal of Public Health.

[CR26] Fricker M (2007). Epistemic injustice: Power & the ethics of knowing.

[CR27] Fritz L, Meinherz F (2020). Tracing power in transdisciplinary sustainability research: An exploration. Gaia.

[CR28] Game ET (2018). Cross-discipline evidence principles for sustainability policy. Nature Sustainability.

[CR29] Ghiara V, Russo F (2019). Reconstructing the mixed mechanisms of health: The role of bio- and socio-markers. Longitudinal and Life Course Studies.

[CR30] Glennan, S., & Illari, P. (Eds.) (2018). *The Routledge handbook of mechanisms and mechanical philosophy*.

[CR31] Glennan S, Illari P, Weber E (2022). Six theses on mechanisms and mechanistic science. Journal for General Philosophy of Science.

[CR32] Good, I. J. (1966). On the principle of total evidence. *British Journal for the Philosophy of Science* 17(4).

[CR33] Haack S (1993). Evidence and inquiry: Towards reconstruction in epistemology.

[CR34] Haack S (2001). Clues to the puzzle of scientific evidence. Principia.

[CR35] Haack S (2011). Defending science-within reason: Between scientism and cynicism.

[CR36] Haack S (2014). Evidence Matters: Science, Proof, and Truth in the Law.

[CR37] Hammersley M (2005). Is the evidence-based practice movement doing more good than harm? Reflections on iain chalmers’ case for research-based policy making and practice. Evidence and Policy.

[CR38] Haraway D (2020). Situated knowledges: The science question in feminism and the privilege of partial perspective. Feminist Theory Reader.

[CR39] Hess DJ, Gross M, McGoey L (2012). Undone science and social movements: A review and typology. The Routledge international handbook of ignorance studies.

[CR40] Hirsch, P., & Brosius, J. (2013). Navigating complex trade-offs in conservation and development: An integrative framework. *Issues in Interdisciplinary Studies*.

[CR41] IPCC, 2023. (2023). IPCC, 2023: Summary for policymakers. In H. Lee, & J. Romero (Eds.), *Climate change 2023: Synthesis report. A report of the Intergovernmental Panel on Climate Change.*

[CR42] Israel BA, Eng E, Schulz AJ, Parker EA (2013). Methods for community based partipatory research for health.

[CR43] Jagosh J, Bush PL, Salsberg J, Macaulay AC, Greenhalgh T, Wong G, Cargo M, Green LW, Herbert CP, Pluye P (2015). A realist evaluation of community-based participatory research: Partnership synergy, trust building and related ripple effects. BMC Public Health.

[CR44] Johnson RB, Russo F, Schoonenboom J (2019). Causation in mixed methods research: The meeting of philosophy, science, and practice. Journal of Mixed Methods Research.

[CR45] Kelly MP, Kelly RS, Russo F (2014). The Integration of social, behavioral, and biological mechanisms in models of pathogenesis. Perspectives in Biology and Medicine.

[CR46] Kelly MP, Russo F (2018). Causal narratives in public health: the difference between mechanisms of aetiology and mechanisms of prevention in non-communicable diseases. Sociology of Health & Illness.

[CR47] Kelly MP, Russo F (2021). The ‘Lifeworld’ of health and disease and the design of public health interventions. MEFISTO Journal of Medicine, Philosophy, and History.

[CR48] Kelty C, Panofsky A (2014). Disentangling Public Participation In Science and Biomedicine. Genome Medicine.

[CR49] Kelty C, Panofsky A, Currie M, Crooks R, Erickson S, Garcia P, Wartenbe M, Wood S (2015). Seven dimensions of contemporary participation disentangled. Journal of the Association for Information Science and Technology.

[CR93] Knickel M, Caniglia G, Knickel K, Šūmane S, Maye D, Arcuri S, Keech D, Tisenkopfs T, Brunori G (2023). Lost in a haze or playing to partners’ strengths? Learning to collaborate in three transdisciplinary European Living Labs. Futures.

[CR50] Kosanic A, Petzold J, Martín-López B, Razanajatovo M (2022). An inclusive future: Disabled populations in the context of climate and environmental change. Current Opinion in Environmental Sustainability.

[CR51] Lagier, D. G. (2020). What is foundherentism, and what can it contribute to the theory of evidence in the law ?* Cosmos + Taxis,**8*(6+7), 44–54.

[CR52] Lang DJ, Wiek A, Bergmann M, Stauffacher M, Martens P, Moll P, Swilling M, Thomas CJ (2012). Transdisciplinary research in sustainability science: Practice, principles, and challenges. Sustainability science.

[CR53] Leask, C. F., Sandlund, M., Skelton, D. A., Altenburg, T. M., Cardon, G., Chinapaw, M. J., De Bourdeaudhuij, I., Verloigne, M., Chastin, S.FM & GrandStand, Safe Step and Teenage Girls on the Move Research Groups (2019). Framework, principles and recommendations for utilising participatory methodologies in the co-creation and evaluation of public health interventions. Research Involvement and Engagement.

[CR54] Ludwig D, El-Hani CN (2020). Philosophy of ethnobiology: Understanding knowledge integration and its limitations. Journal of Ethnobiology.

[CR55] Ludwig, D., Koskinen, I., Mncube, Z., Poliseli, L., & Reyes-Galindo, L. (Eds.) (2021). *Global epistemologies and philosophies of science*. Routledge.

[CR56] Luederitz C, Schäpke N, Wiek A, Lang DJ, Bergmann M, Bos JJ, Burch S, Davies A, Evans J, König A, Farrelly M, Forrest N, Frantzeskaki N, Gibson R, Kay B, Loorbach D, McCormick K, Parodi O, Rauschmayer F, Schneidewind U, Stauffacher M, Stelzer F, Trencher G, Venjakob J, Vergragt P, von Wehrden H, Westley FR (2017). Learning through evaluation–A tentative evaluative scheme for sustainability transition experiments. Journal of Cleaner Production.

[CR57] Malmer, P., Masterson, V., Austin, B., Tengö, M., Sutherland, W. J., Brotherton, P. N. M., Davies, Z.G., Ockendon, N., Pettorelli, N., & Vickery, J. A. (2020). Mobilisation of indigenous and local knowledge as a source of useable evidence for conservation partnerships. *Conservation Research, Policy and Practice*, 82–113.

[CR58] Mann-Jackson L, Alonzo J, Garcia M, Trent S, Bell J, Horridge DN, Rhodes SD (2021). Using community-based participatory research to address STI/HIV disparities and social determinants of health among young GBMSM and transgender women of colour in North Carolina, USA. Health & Social Care in the Community.

[CR59] Menatti L, Bich L, Saborido C (2022). Health and environment from adaptation to adaptivity: A situated relational account. History and Philosophy of the Life Sciences.

[CR60] Meyer, I. H., & Northridge, M. E. (Eds.) (2007). *The health of sexual minorities: Public health perspectives on lesbian, gay, bisexual and transgender populations*. Springer.

[CR61] Minkler, M., Victoria Vásquez, B., Chang, C., & Miller, J. (2010). Policy link. *Community-Based Participatory Action Research.*

[CR62] Montuschi E (2017). Using science, making policy: What should we worry about?. European Journal for Philosophy of Science.

[CR63] Olsson, E. (2021). Coherentist theories of epistemic justification. In *Stanford encyclopedia of philosophy*.

[CR64] Parkhurst J (2017). The politics of evidence: From evidence-based policy to the good governance of evidence.

[CR65] Parkkinen, V. P., Wallmann, C., Wilde, M., Clarke, B., Illari, P., Kelly, M. P., Norell, C., Russo, F., Shaw, B., & Williamson, J. (2018). How to consider evidence of mechanisms: An overview. *Evaluating Evidence of Mechanisms in Medicine: Principles and Procedures*, 23–33. 31314227

[CR66] Petticrew M, Roberts H (2003). Evidence, hierarchies, and typologies: Horses for courses. Journal of Epidemiology and Community Health.

[CR67] Pohl C, Klein JT, Hoffmann S, Mitchell C, Fam D (2021). Conceptualising transdisciplinary integration as a multidimensional interactive process. Environmental Science & Policy.

[CR68] Reed M, Meagher L, Boaz A, Davies H, Fraser A, Nutley S (2019). Using Evidence in Environmental and Sustainability Issues. What works now? Evidence-informed policy and practice.

[CR69] Rhodes SD, Ballard PJ, Moore KR, Klein K, Randall I, Lischke M, Vissman AT, Lengerich EJ, Daniel SS, Skelton JA (2021). Community-engaged research in translational science: Innovations to improve health in appalachia. Journal of Clinical and Translational Science.

[CR70] Rhodes SD, Daniel-Ulloa J, Wright SS, Mann-Jackson L, Johnson DB, Hayes NA, Valentine JA (2021). Critical elements of community engagement to address disparities and related social determinants of health: The centers of disease control and prevention community approaches to reducing sexually transmitted disease initiative. Sexually Transmitted Diseases.

[CR71] Rhodes SD, Malow RM, Jolly C (2012). Community-based participatory research (CBPR): A new and not-so-new approach to HIV/AIDS prevention, care, and treatment. AIDS Education and Prevention: Official Publication of the International Society for AIDS Education.

[CR72] Robinson, C. J., Maclean, K., Hill, R., Bock, E., & Rist, P. (2016). Participatory mapping to negotiate indigenous knowledge used to assess environmental risk. *Sustainability Science*

[CR73] Russo F (2022). Techno-scientific practices: An informational approach.

[CR74] Russo F, Hirsch P (2023). Navigating complex trade-offs in public health interventions. Journal of Evaluation in Clinical Practice.

[CR75] Rütten, A., Semrau, J., & Wolff, A. R. (2023). Entwicklung gesundheitsförderlicher Strukturen durch kooperative Planung. *Prävention und Gesundheitsförderung*.

[CR76] Sanderson I (2002). Evaluation, policy learning and evidence-based policy making. Public Administration.

[CR77] Sanderson I (2006). Complexity, ‘practical rationality’ and evidence-based policy making. Policy & Politics.

[CR78] Smith BM, Chakrabarti P, Chatterjee A, Chatterjee S, Dey UK, Dicks LV, Giri B, Laha S, Majhi RK, Basu P (2017). Collating and validating indigenous and local knowledge to apply multiple knowledge systems to an environmental challenge: A case-study of pollinators in India. Biological Conservation.

[CR79] Sterner T, Barbier EB, Bateman I, van den Bijgaart I, Crépin AS, Edenhofer O, Fischer C, Habla W, Hassler J, Johansson-Stenman O, Lange A, Polasky S, Rockström J, Smith H, Steffen W, Wagner G, Wilen J, Alpízar F, Azar C, Carless D, Chávez C, Coria J, Engström G, Jagers SC, Köhlin G, Löfgren Å, Pleijel H, Robinson A (2019). Policy design for the anthropocene. Nature Sustainability.

[CR80] Stirling A (2010). Keep it complex. Nature.

[CR81] Tengö M, Brondizio ES, Elmqvist T, Malmer P, Spierenburg M (2014). Connecting diverse knowledge systems for enhanced ecosystem governance: The multiple evidence base approach. Ambio.

[CR82] Tengö M, Hill R, Malmer P, Raymond CM, Spierenburg M, Danielsen F, Elmqvist T, Folke C (2017). Weaving knowledge systems in IPBES, CBD and beyond—lessons learned for sustainability. Current Opinion in Environmental Sustainability.

[CR83] Turnhout, E., Van Bommel, S., & Aarts, N. (2010). How participation creates citizens: Participatory governance as performative practice. *Ecology and Society**15*(4).

[CR84] Turnhout E, Metze T, Wyborn C, Klenk N, Louder E (2020). The politics of co-production: Participation, power, and transformation. Current Opinion in Environmental Sustainability.

[CR85] Valles S (2018). Philosophy of population health: Philosophy for a new public health era.

[CR86] Venkatapuram S (2011). Health justice: An Argument from the capabilities approach.

[CR87] Walker SC, Whitener R, Trupin EW, Migliarini N (2015). American Indian perspectives on evidence-based practice implementation: Results from a statewide tribal mental health gathering. Administration and Policy in Mental Health and Mental Health Services Research.

[CR88] Wallerstein N, Duran B (2010). Community-based participatory research contributions to intervention research: The intersection of science and practice to improve health equity. American Journal of Public Health.

[CR89] Walls M, Chambers R, Begay M, Masten K, Aulandez K, Richards J, Gonzalez M, Forsberg A, Nelson L, Larzelere F, McDougall C, Lhotka M, Grass R, Kellar S, Reid R, Barlow A (2022). Centering the Strengths of American Indian Culture, families and communities to overcome type 2 diabetes. Frontiers in Public Health.

[CR90] West S, van Kerkhoff L, Wagenaar H (2019). Beyond ‘linking knowledge and action’: Towards a practice-based approach to transdisciplinary sustainability interventions. Policy Studies.

[CR91] Wyborn C, Datta A, Montana J, Ryan M, Leith P, Chaffin B, Miller C, Van Kerkhoff L (2019). Co-producing sustainability: Reordering the governance of science, policy, and practice. Annual Review of Environment and Resources.

